# Characterization and evaluation of mycosterol secreted from endophytic strain of *Gymnema sylvestre* for inhibition of α-glucosidase activity

**DOI:** 10.1038/s41598-019-53227-w

**Published:** 2019-11-21

**Authors:** Amit Ranjan, Rajesh Kumar Singh, Saumya Khare, Ruchita Tripathi, Rajesh Kumar Pandey, Anurag Kumar Singh, Vibhav Gautam, Jyoti Shankar Tripathi, Santosh Kumar Singh

**Affiliations:** 1 Centre of Experimental Medicine and Surgery, Institute of Medical Sciences, Banaras Hindu University, Varanasi, 221 005 India; 2 Department of Kayachikitsa, Faculty of Ayurveda, Institute of Medical Sciences, Banaras Hindu University, Varanasi, 221 005 India; 3 Department of Dravyaguna, Faculty of Ayurveda, Institute of Medical Sciences, Banaras Hindu University, Varanasi, 221 005 India; 4 Department of Biochemistry, Institute of Science, Banaras Hindu University, Varanasi, 221 005 India

**Keywords:** Drug discovery and development, Applied microbiology

## Abstract

Endophytic fungi produce various types of chemicals for establishment of niche within the host plant. Due to symbiotic association, they secrete pharmaceutically important bioactive compounds and enzyme inhibitors. In this research article, we have explored the potent α-glucosidse inhibitor (AGI) produced from *Fusarium equiseti* recovered from the leaf of *Gymnema sylvestre* through bioassay-guided fraction. This study investigated the biodiversity, phylogeny, antioxidant activity and α-glucosidse inhibition of endophytic fungi isolated from *Gymnema sylvestre*. A total of 32 isolates obtained were grouped into 16 genera, according to their morphology of colony and spores. A high biodiversity of endophytic fungi were observed in *G*. *sylvestre* with diversity indices. Endophytic fungal strain *Fusarium equiseti* was identified through DNA sequencing and the sequence was deposited in GenBank database (https://ncbi.nim.nih.gov) with acession number: MF403109. The characterization of potent compound was done by FTIR, LC-ESI-MS and NMR spectroscopic analysis with IUPAC name 17-(5-ethyl-6-methylheptan-2-yl)-10,13-dimethyl-2,3,4,7,8,9,10,11,12,13,14,15,16,17-tetradecahydro-1H-cyclopenta[a] phenanthren-3-ol. The isolated bioactive compound showed significant α-amylase and α-glucosidase inhibition activity with IC_50_ values, 4.22 ± 0.0005 µg/mL and 69.72 ± 0.001 µg/mL while IC_50_ values of acarbose was 5.75 ± 0.007 and 55.29 ± 0.0005 µg/mL respectively. This result is higher in comparison to other previous study. The enzyme kinetics study revealed that bioactive compound was competitive inhibitor for α-amylase and α-glucosidase. *In-silico* study showed that bioactive compound binds to the binding site of α-amylase, similar to that of acarbose but with higher affinity. The study highlights the importance of endophytic fungi as an alternative source of AGI (*α*-glucosidase inhibition) to control the diabetic condition *in vitro*.

## Introduction

Diabetes mellitus (DM) is a multifactorial metabolic disease characterized by hyperglycemia. In 2017, approximately, 425 million people (1 in 11 adults) affected with diabetes and it may rise to 693 million in 2045. Diabetes mellitus is the fourth leading cause of NCD deaths (1.5 million or one death every eight seconds)^1,2^. Diabetes mellitus is a metabolic disorder which is influenced by environmental and genetic factors. It is not curable till date therefore, diabetic patient maintain their blood glucose level by both exercise and medications. At present time, various groups of synthetic drugs are used in diabetes. The α-glucosidase inhibitors (AGI) are a specific class of antidiabetic drug that could reduce the blood glucose level by delay of digestion of carbohydrate. It is most effective with other class of synthetic antidiabetic drugs which may cause severe side effects, and indicates the need for new bioactive molecules with fewer side effects. Secondary metabolites offer inexpensive, safe and less side effect way for the treatment of diabetic patients. Approx, 80% of worlds population depends on the natural remedies for the treatment. However, traditionally used medicinal plants have served as a rich resource of drug due to their vast chemical diversity but harvesting on large scale may cause massive depletion of biodiversity.

Endophytic products provide opportunities for the search of new drug. Endophytes are present inside the plant tissues without causing any negative symptoms to the host plant^3^. It may be fungi, bacteria and actinomycetes. Endophytes and their host plant make symbiotic association in which both are benefited by horizontal transfer of genetic information. This association helps in the synthesis of broad range of natural active compounds^4^. Numerous fungal endophytes have been identified in last two decades from approximate 300,000 plants^5^; these fungal endophytes reside in all parts of the plant (stem, root, leaf, fruit, flower, and seed). Some of them produce bioactive compounds, although very few of them have been studied^6^. The discovery of the billion-dollar anti-cancer drug, taxol, isolated from endophytic fungus *Taxomyces andreanae* of the plant *Taxus brevefolia*^7^ started the new era of drug discovery from endophytic fungi. Endophytes have been identified as the promising source of novel bioactive compounds of pharmacological importances^8^ such as anticancer cajanol^9^, antimycotics steroid 22-triene-3β-ol^10^, anti-inflammatory ergoflavin^11^, podophyllotoxin and kaempferol^12^, antioxidant lectin^13^, cytotoxic radicicol^14^ and immunosuppressive sydoxanthone A, B^15^.

The fungal endophytes are the repository of novel secondary metabolites. At present time, many researchers have studied some natural product from various medicinal plants and microorganisms having potential to inhibit α-glucosidase activity^16^. The medicinal plant *Gymnema sylvestre* is an indigenous herb which belongs to *Asclepiadaceae* family, native to India, South Asia and Africa^17^. In reference survey and analysis, it is found that 96 medicinal plant species were showing mutualism; meaning mutual benefits in terms of the fungus-host relationships and these species were distributed among 46 families, including *Asclepiadaceae* (1 taxon)^5^. This plant is distinguished for the treatment of diabetes in India for over 2000 years^18^. The antidiabetic property of Gymnema Sylvestre is mentioned in Vedic literature and the Ayurvedic Pharmacopoeia of India (Part 1; Vol. V). This plant also inhibits glucose absorption from the intestine^19^ and is used for many polyherbal formulations, leading to extinction of this medicinal plant. The bioactive compounds and various polyherbal formulation of this plant plays an important role in many diseases but little work is reported on their endophytes. In this study, the fungal endophytes associated with *G. Sylvestre* have been studied as an alternative source of antidiabetic drug. The current study reports for the first time *Fusarium equiseti* (Acc. No. MF 403109) isolated from *G. sylvestre* (Acc. No. DG/18/172) which produces mycosterol with α-glucosidase inhibitory activity. This result offers an opportunity for further investigation and utilization of endophytic fungi associated with *G*. *sylvestre*. Hence, the study highlights the importance of AGI as endophytic mediated control to diabetes.

## Results

### Biodiversity of endophytes isolated from *Gymnema sylvestre*

**Figure 1:**
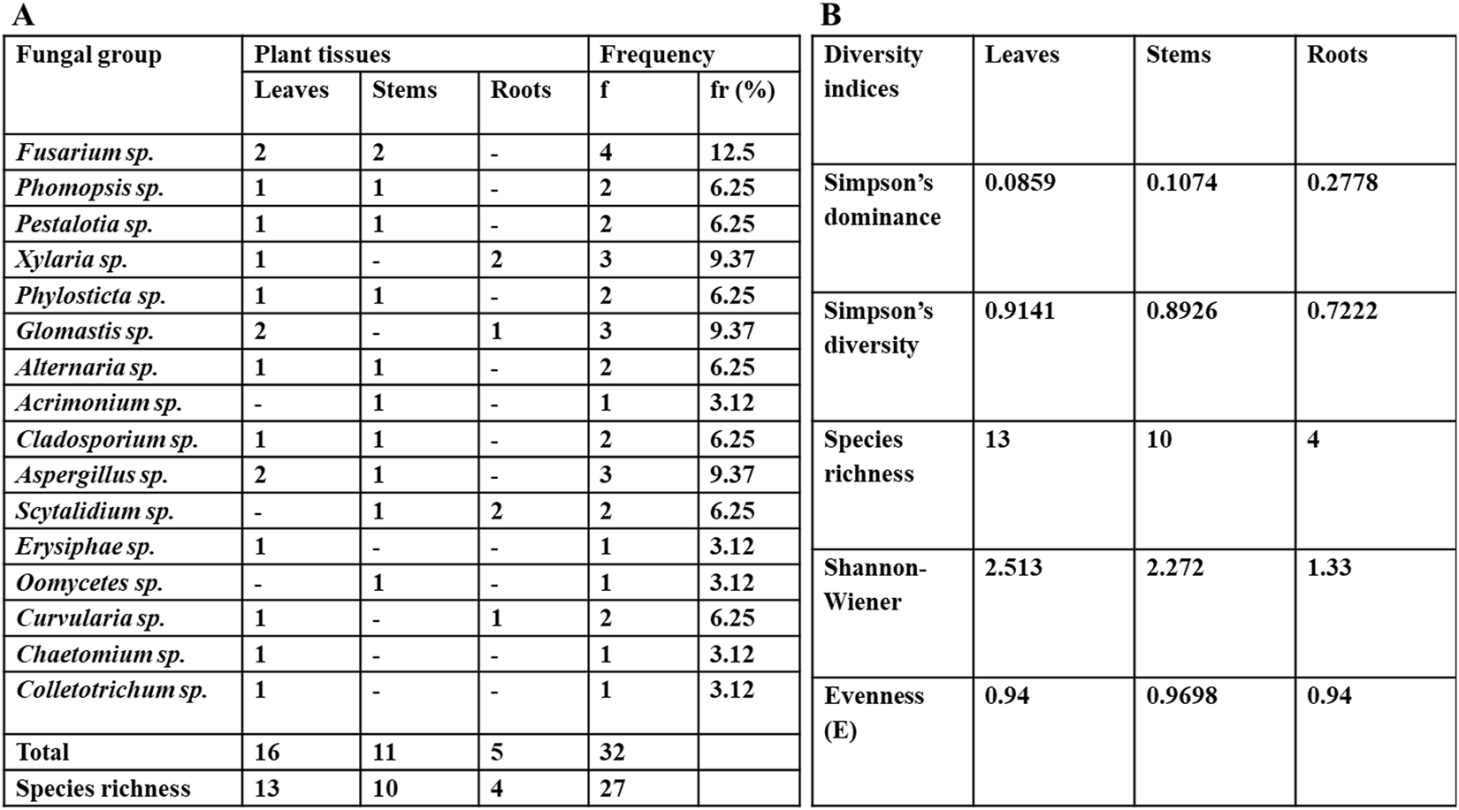
(**A**) List of endophytic fungi obtained from the medicinal plant *G. sylvestre*. (**B)** Diversity indices of endophytic fungi isolated from different parts of *G. sylvestre*.

Different parts of a medicinal herb *G. sylvestre* were explored for fungal endophytes and total 16 fungal groups were isolated. A total of 32 fungal isolates of which 16 isolated from leaf, 11 from stem and 5 were isolated from the root of *G. sylvestre*. Strains were identified on the basis of culture characteristic, microscopic studies and spore morphology. A relative frequency of *Fusarium* sp. was found to be highest while three groups *Xylaria* sp., *Glomastis* sp., *Aspergillus* sp. were found to be in moderate range and remaining were in low frequency (Fig. 1A). Species richness was found to be highest in leaves in comparison to other parts of the plant.

The biodiversity of fungal endophytes isolated from different tissues were evaluated by various diversity indices such as Simpson’s diversity index (1-D), Simpson’s dominance index, Species richness (Dmn), Shannon-Wiener index (H) and Evenness. The highest tissue-specific fungal dominance was found in the root (0.2778) then in stem (0.1074) and least in leaf (0.0859). *Fusarium* sp. was most dominant which was isolated from leaves and stems. The Shannon and Simpson’s indices, respectively, indicated consistency and a high certainty of endophytic fungal species in the root (1.33). Species richness indicates highly diverse and taxonomically rich fungal endophytes i.e. in leaves (13). Species evenness is uniform in leaves and roots while it is slightly higher (0.96) in stems. These diversity indexes represent the significant of endophytes within and between the different tissues of *G. sylvestre* (Fig. 1B).

### Screening of endophytes for antidiabetic activity and bioactivity guided fractionation

After isolation of fungal endophytes from *G*. *Sylvestre*, we initiated comprehensive screening to find the potent fungal endophytes having antidiabetic activity such as α-amylase and α-glucosidase inhibitors. Broth cultures of different fungal isolates were evaluated in different organic solvent to find the antidiabetic activity and suitable organic solvent for further study.

Among 32 isolates, one *Fusarium* sp. extracted in ethyl acetate and chloroform was found as active inhibitor of porcine pancreas α-amylase (EC 3.2.1.1) and α-glucosidase (EC 3.2.1.20) from *Saccharomyces cerevisiae*. Those isolate having antidiabetic activity were selected to identify and characterize the bioactive compound. A total of 32 fungal isolates of *G*. *sylvestre* of which one isolate of *Fusarium* sp. isolated from leaf tissue of *G*. *sylvestre* was recorded as an incidental rare strain (1/32 isolates).

The chloroform soluble fraction obtained through silica gel vacuum liquid chromatography was more active than ethyl acetate extract. Potent fraction of chloroform extract of *F*. *equiseti* was refractionated through HPTLC. Five separate fractions were obtained of which one sub fraction exhibited high α-amylase and α-glucosidase inhibition with IC_50_ values, 4.22 ± 0.0005 and 69.72 ± 0.001 µg/mL respectively. While IC_50_ values of acarbose against α-amylase and α-glucosidase were 5.75 ± 0.007 and 55.29 ± 0.0005 µg/mL respectively.

### Identification and chacterization of potent antidiabetic endophytic strain

**Figure 2:**
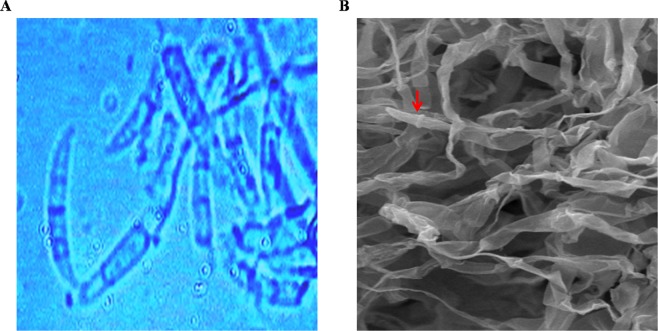
(**A**) Light microscopy image of potent fungal strain. **(B)** SEM images for potent fungal strain indicating the morphology (half-moon shaped) of the spores.

**Figure 3:**
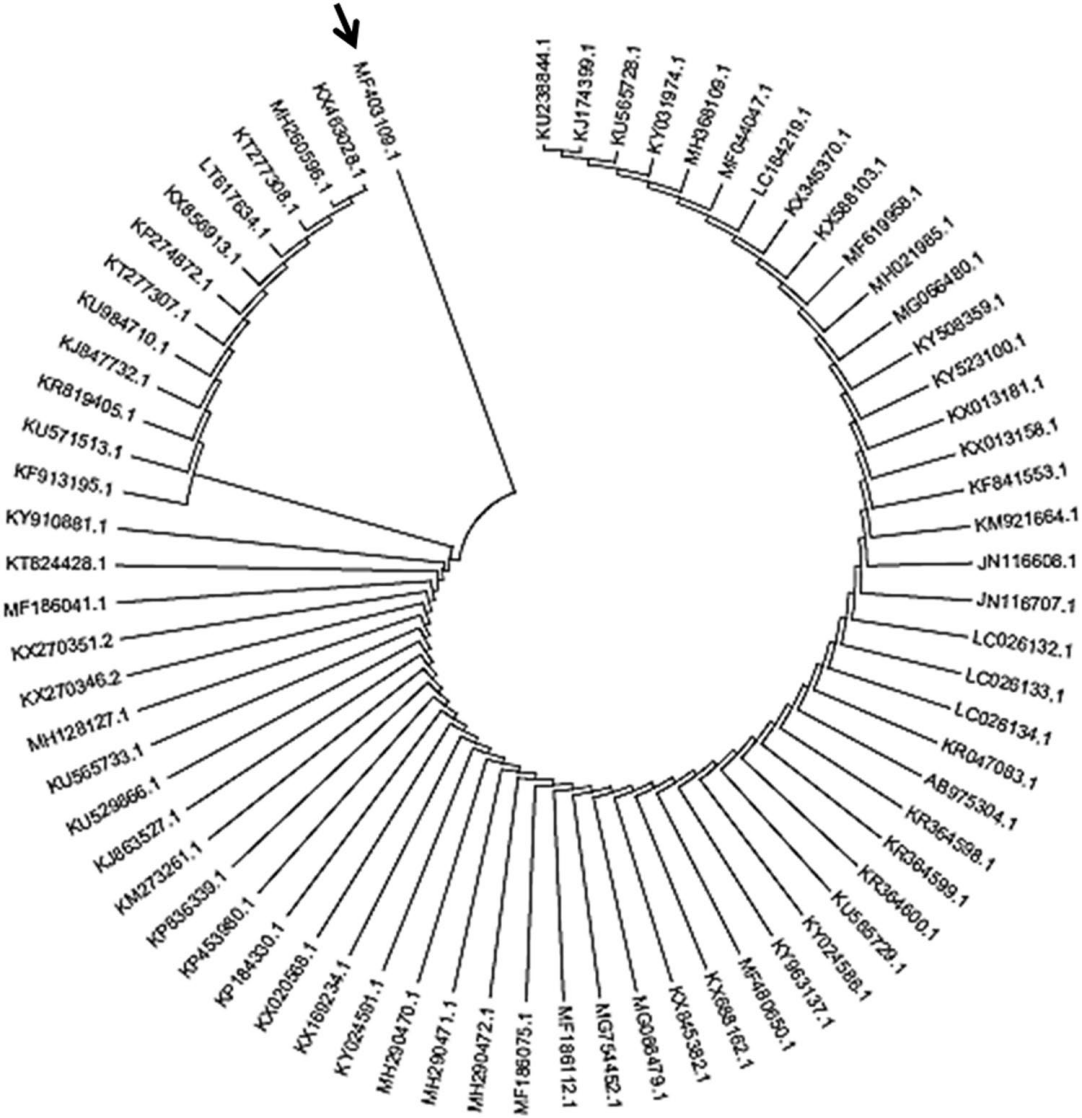
Phylogenetic tree of the identified strain with other *Fusarium* species using MEGA6.

The morphological identification was done by microscopic studies, culture characteristics and spore morphology (Fig. 2A,B). The molecular identification was done by DNA sequencing. The obtained fungal sequence was deposited in GeneBank database (https://ncbi.nim.nih.gov) with accession number MF 403109. The phylogenetic analysis involved 70 nucleotide sequences of *Fusarium* sp., phylogenetic tree was constructed using NJ based ITS sequences with more than 92% similarity. The maximum likelihood estimate of gamma parameter for site rates was done with MEGA6. A high degree of genetic diversity among *Fusarium sp*. was observed in phylogenetic analysis (Fig. 3). A potent fungal strain *Fusarium equiseti* ‘SKS01’ was isolated from the leaf of *G. sylvestre*.

### Chemical characterization of α-glucosidse inhibitor (AGI)

**Figure 4:**
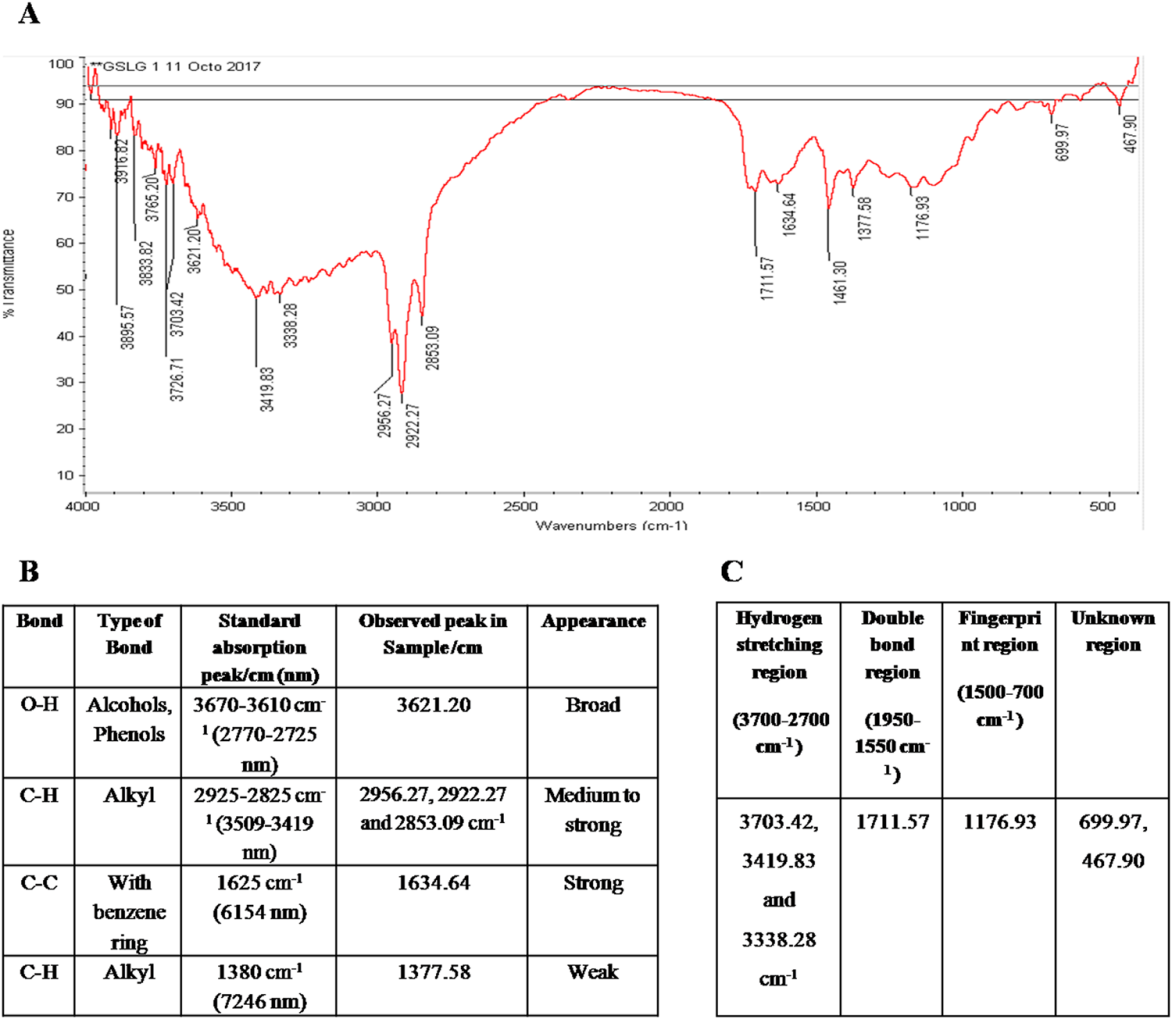
(**A**) FTIR spectrum for mycosterol produced by *F. equiseti*. **(B)** Table showing the FTIR spectroscopy peaks and functional groups detected in sample of *F. equiseti*. **(C)** Table showing the FTIR peaks of sample of *F. equiseti* obtained in different regions.

In IR spectrum, peak showed O-H Stretching vibrations at 3621.2 cm^−1^ which represent alcoholic group however, three peaks were obtained in hydrogen stretching region (3703.42, 3419.83 and 3338.28 cm^−1^). Three medium to strong peaks were obtained at 2956.27, 2922.27 and 2853.09 cm^−1^ were due to aliphatic C-H vibrations, fall in between region 2925 and 2850 cm^−1^ (Fig. 4A). The double bond region (1950–1550 cm^−1^) -C=O stretching vibration is characterized by the absorption at 1711.57 cm^−1^, indicating the presence of double bond in cyclohexane. C-C stretching vibrations occurred at 1634.64 while peak 1461.30 indicate aliphatic structure with bending vibration, assigned as alkane while peak at 1176.93 cm^−1^ indicate amine/ester/tertiary alcohol and vinyl group (Fig. 4B,C).

**Figure 5:**
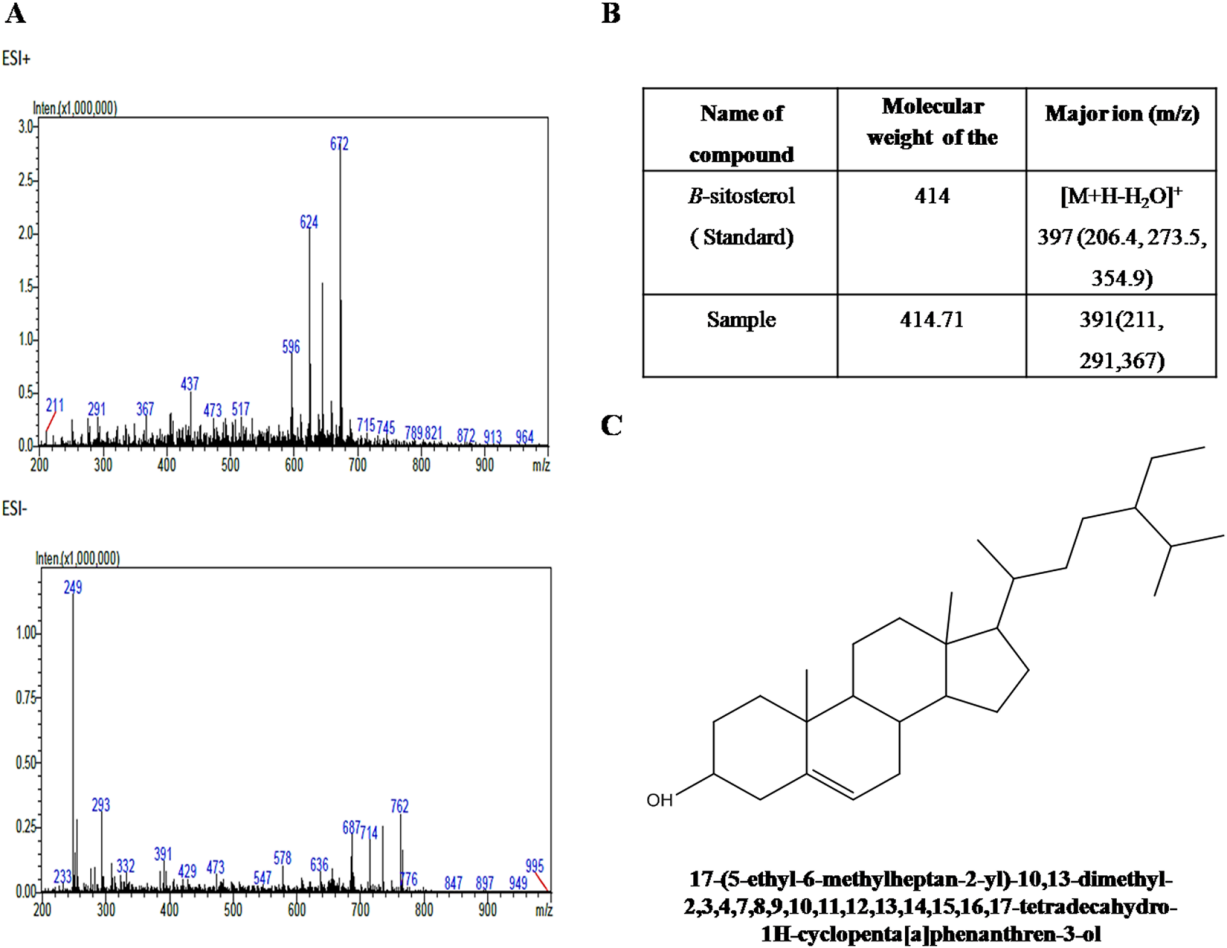
(**A**) LC-ESI-MS spectrum of mycosterol obtained from *F*. *Equiseti*. **(B)** Comparison between characteristic ions of compound isolated from *F. equiseti* and *β-*sitosterol. **(C)** Structure and IUPAC name of mycosterol obtained from *F. equiseti*. **(D)** Parameters of enzyme kinetics against α-amylase and α-glucosidase.

Electrospray ionization mass (HR-ESI-MS) analysis of compound on high resolution demonstrated a pseudomolecular ion peak at (*m/z*) 391, representing the molecular formula as C_29_H_50_O. This analysis indicated the molecular mass of the compound as 414.71 m/z g/mol with Pubchem CID as 12303662 (Fig. 5A,B)

^1^H NMR (500 MHz, CDCL_3_Bruker, Switzerland), δH: 1.378–0.901 (-CH_3_), δH: 3.334 (-CH_2_), δH: 1.582 (alcoholic –OH) *δ = *7.285–7.231 (Ar-H); *δ = *3.559–3.514 (aliphatic CH) were detected and the structure was further confirmed by ^13^C NMR spectroscopy.

Potent fraction of chloroform extract of *F. equiseti* showed characteristic of β-sitosterol. This mycosterol derivative showed the similar characteristic appeared in phytosterol such as β-sitosterol, campestrol and stigmosterol^20^. Identity of this purified compound was confirmed by FTIR, LC-ESI-MS and NMR spectroscopic analysis and their chemical structures are shown in Fig. 5C and its IUPAC name is 17-(5-ethyl-6-methylheptan-2-yl)-10,13-dimethyl-2,3,4,7,8,9,10,11,12,13,14,15,16,17-tetradecahydro-1H-cyclopenta[a]phenanthren-3-ol.

### *In-vitro* study of the plant extract and mycosterol

The sub fraction of extract of medicinal plant *G. sylvestre* exhibited α-amylase and α-glucosidase inhibition with IC_50_ values, 10.47 ± 0.0005 and 85.73 ± 0.001 µg/mL respectively. The potent subfraction of chloroform extract of *F*. *equiseti* exhibited high α-amylase and α-glucosidase inhibition with IC_50_ values, 4.22 ± 0.0005 µg/mL and 69.72 ± 0.001 µg/mL respectively. The IC_50_ values of acarbose was 5.75 ± 0.007 and 55.29 ± 0.0005 µg/mL respectively (Table 1).

**Table 1:** Parameters of enzyme kinetics against α-amylase and α-glucosidase.

Enzyme Inhibition	Inhibitory activity of sub fraction of	IC_50_ value (µg/mL)	V_max_ (µmoles min^−1^ mg^−1^) K_m_ (mg/mL)	Type of Inhibition
α- amylase	Medicinal plant *G. sylvestre extract*	10.47 ± 0.0005	495.0, 0.525	Uncompetitive
	Endophytic strain *F. equiseti extract*	4.22 ± 0.0005	590.0, 0.80	Competitive
	Acarbose (Inhibitor)	5.75 ± 0.007	590, 0.74	Competitive
α- glucosidase	*G. sylvestre extract*	85.73 ± 0.001	1290, 0.84	Non-competitive
	*F. equiseti extract*	69.72 ± 0.001	1240, 0.73	Uncompetitive
	Acarbose (Inhibitor)	55.29 ± 0.0005	1590, 1.22	Competitive

### Enzyme kinetics of active fraction of *G*. *sylvestre* and mycosterol

**Figure 6:**
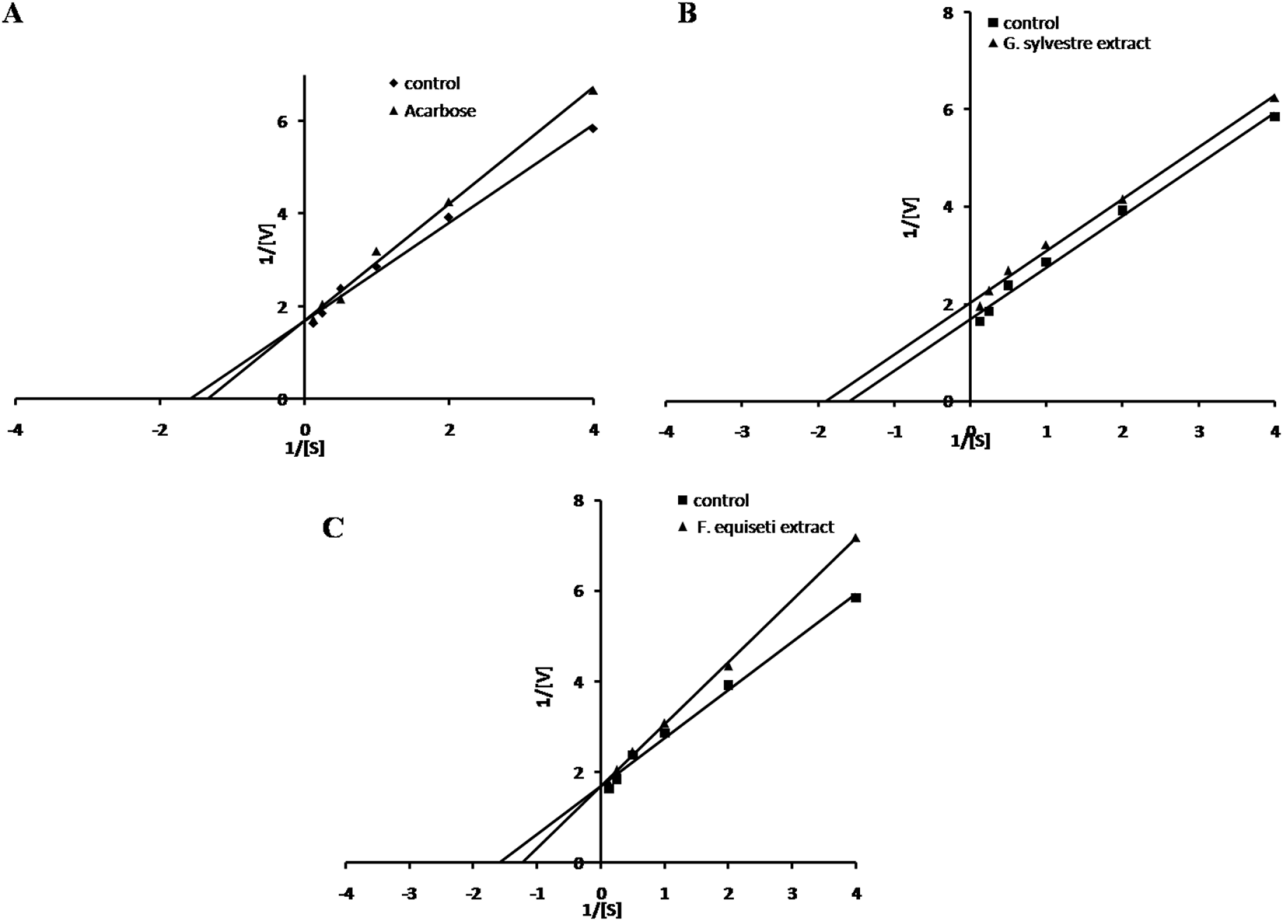
Lineweaver–Burk plots of α-amylase inhibition by **(A)** Acarbose, **(B)** Extract of *G. sylvestre* and **(C)** Mycosterol of *F. equiseti*.

**Figure 7:**
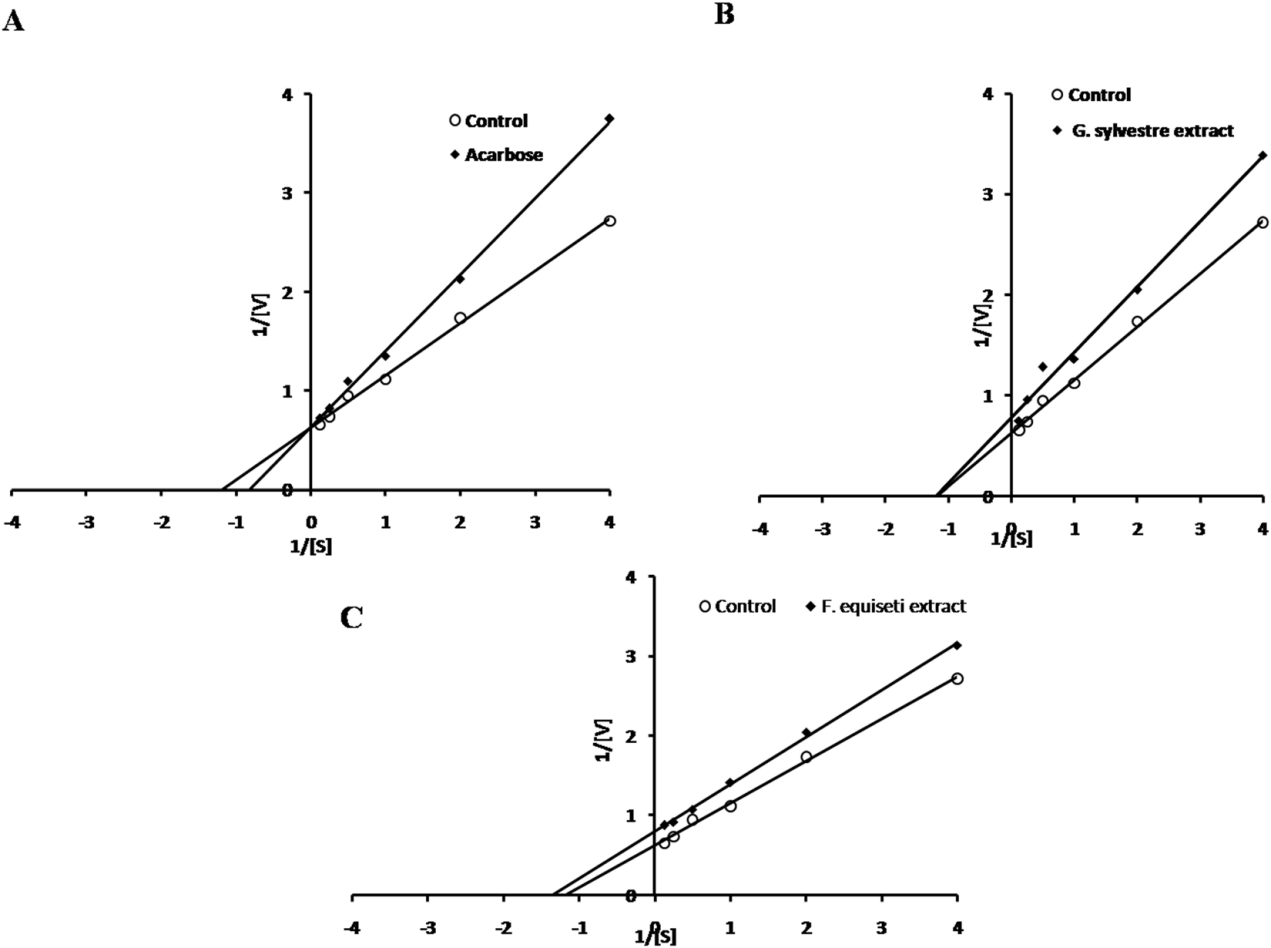
Lineweaver–Burk plots of α-glucosidase inhibition by **(A)** Acarbose, **(B)** Extract of *G. sylvestre* and **(C)** Mycosterol of *F. equiseti*.

The mode of inhibition of α-amylase and α-glucosidase was determined using the extract of *G*. *sylvestre* and mycosterol from *F*. *equiseti* using Lineweaver–Burk plot as shown in Figures 6 and 7. The present study revealed mycosterol derived from bioactive subfraction of *F*. *equiseti* as competitive inhibitor of α-amylase which binds strongly to active site of enzyme while uncompetitive inhibitor of α- glucosidase which is likely to inhibit through binding enzyme-substrate complex. The extracts of *G*. *sylvestre* also showed uncompetitive inhibition of α-amylase but appeared as non-competitive for α-glucosidase. The non-competitive inhibitor is likely to show inhibition by binding to the allosteric site of enzyme other than the active site. Moreover, acarbose was found to be the competitive inhibitor in case of both the enzymes and was used as a reference standard. The kinetics of enzyme (Vmax, Km), IC_50_ value for the inhibitor and their mode of inhibition was studied using Lineweaver Burk pot. The Km value for starch as the substrate was found to be 0.85 (mM) and Vmax 590.40 (µMmin^−1^) in case of α-amylase. In addition, Km value for p-NPG as the substrate was found to be 2.82 (mM) and Vmax 150.62 (µMmin^−1^) in case of α-glucosidase.

### Cytotoxicity assay

**Figure 8:**
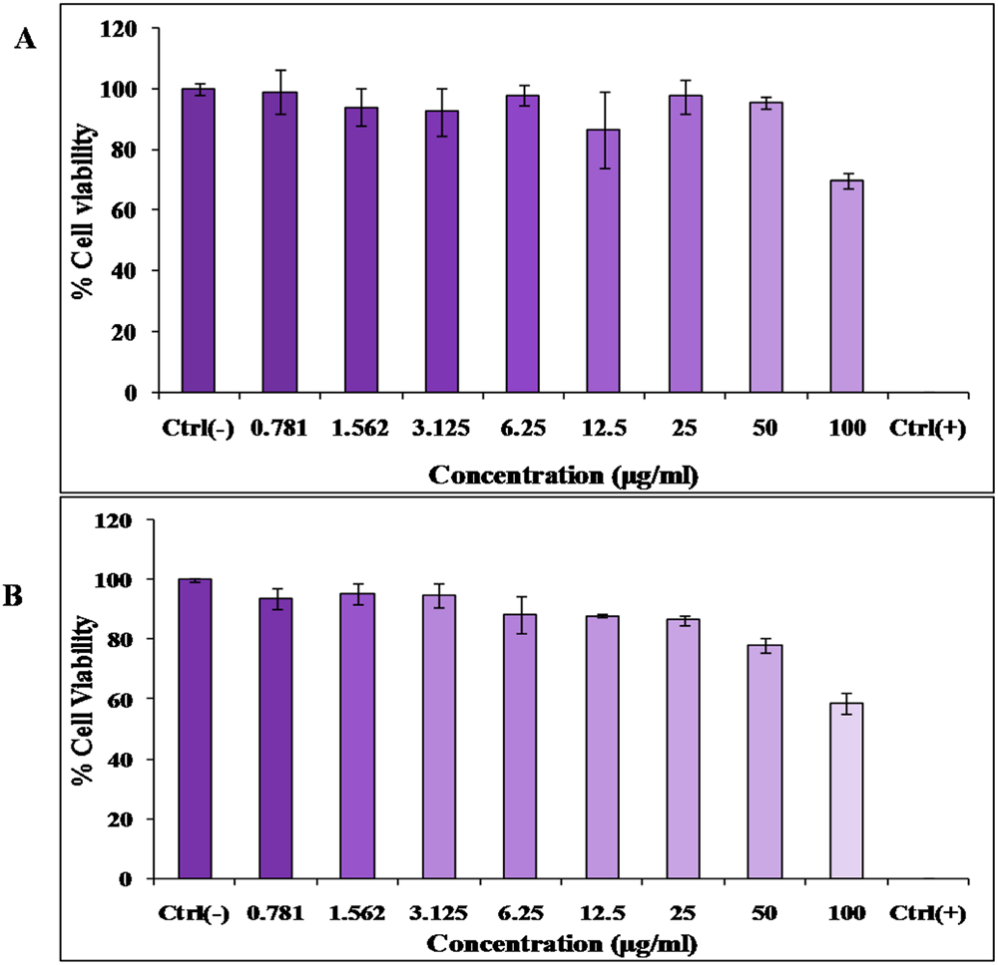
(**A**) Cytotoxicity of extract of *G. sylvestre* on L929 cells (mouse fibroblast cell line). **(B)** Cytotoxicity of bioactive subfraction derivative of *F. equiseti* on L929 cells.

The cytotoxicity study of bioactive fraction of plant extract and mycosterol of fungus was done using L929 cells through MTT assay to evaluate the toxicity. The cytotoxicity assay showed that the bioactive fraction of plant extract and mycosterol of fungus had very low cytotoxicity over a concentration range of 0 to 400 μg/mL. Cellular viability was minimally affected even at high concentration (70% cellular viability at 100 μg/mL for bioactive fraction of plant extract and 78.15% at 50 μg/mL for mycosterol of fungus) (Fig. 8).

### *In-silico* study

**Figure 9:**
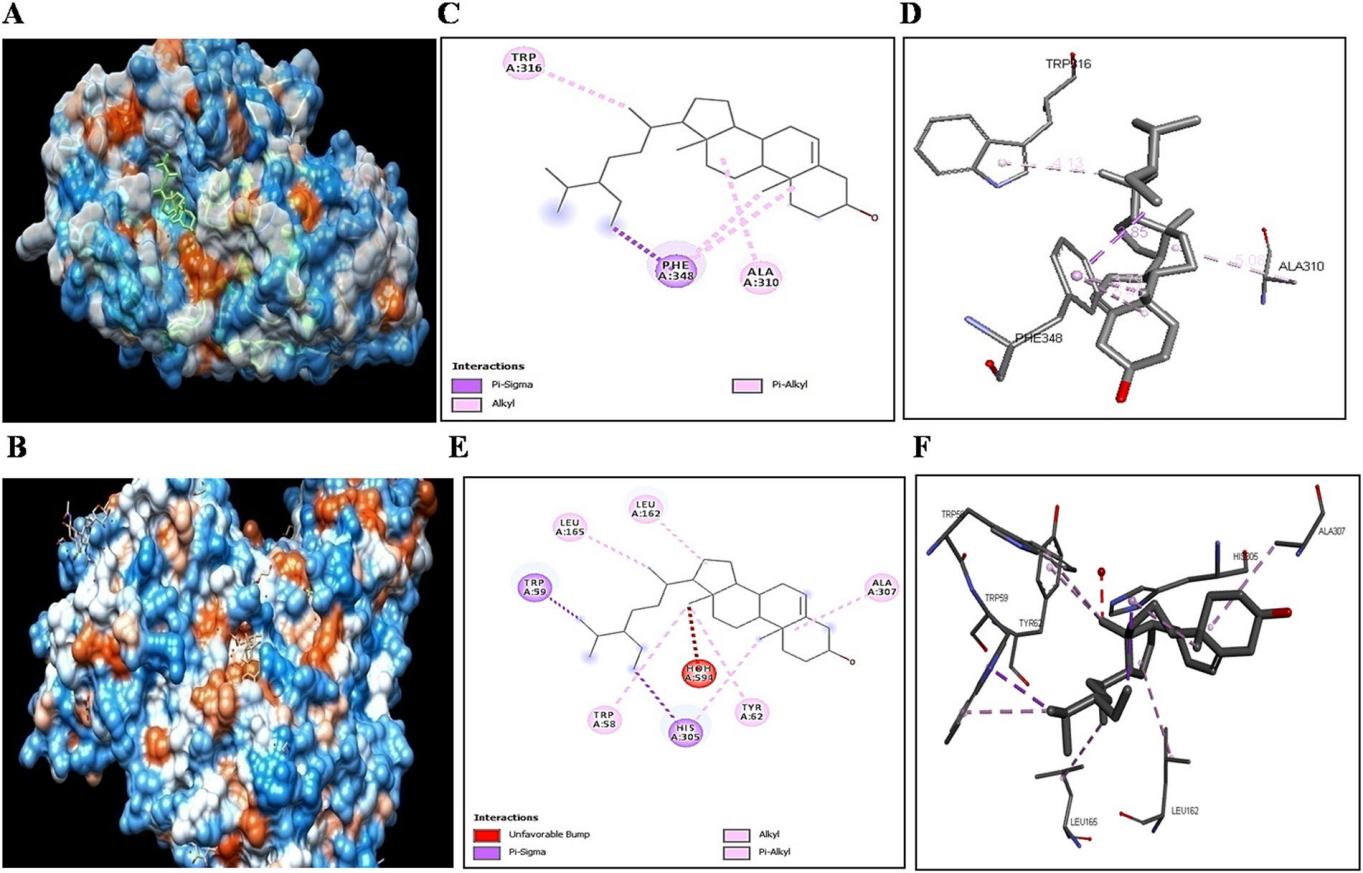
Binding of bioactive compound to α-amylase and α-glucosidase active pocket. **(A)** Docked conformation of mycosterol with human pancreatic α-amylase. **(B)** Docked conformation of mycosterol with human α-glucosidase. **(C,D)** Visualization of mycosterol complexed with α-amylase, shown in stick and line representation **(C)** 2 D structure; **(D)** 3 D structure. **(E,F)** Visualization of mycosterol complexed with α-glucosidase, shown in line and stick representation. **(E)** 2 D structure of mycosterol complexed with α-glucosidase in line representation. **(F)** 3D structure of mycosterol complexed with α-glucosidase in stick representation.

Molecular docking studies provided information for the binding of mycosterol to α-amylase and α -glucosidase binding site (Fig. 9A,B). In molecular docking analysis, it was found that hydrophobic amino acid (Trp:316, Phe:348 and Ala :310) in catalytic site of α-amylase make strong bonding with potent compound (Fig. 9C,D). The conservative residue (Trp:58, Trp:59) and charge residue (Leu:162) of α-glucosidase enzymes indicates strong hydrophobic interaction with potent compound while polar residue (Tyr:62, His:305) and Ala:307 make less hydrophobic interaction to ligand (Fig. 9E,F). The potent compound interaction with α-amylase showed better interaction and was conferred with highest interface area size (554.10), geometric score (5074) and desolvation energy ACE (−75.54). While potent compound interaction with α-glucosidase was also conferred with highest geometric score (5334), interface area size (710.20) and desolvation energy ACE (−290.14).

## Discussion

Endophytes are ‘the tiny factories of nature’ which produce various bioactive secondary metabolites and has a capability to encode similar type of metabolites as synthesized by their host plant^21^. These bioactive compounds have a broad spectrum of biological activities such as antimicrobial, antioxidant, antidiabetic and anticancer. These bioactive compounds are classified into many categories such as alkaloids, phenol, steroids, terpenoids and lignin^8^. Some steroid derivative showed hypoglycaemic activity by decreasing the blood glucose levels in diabetic rat^22^.

The richness of endophytes depends upon various factors of environment and habitat. In previous study, 11 fungal groups were isolated in summer session^3^. In winter session, out of 16 fungal groups, one potent fungal strain *F. equiseti* (MF 403109) was deposited in NCBI Genebank and published in Pubmed. Phylogenetic analysis indicates that potent strain is ancient in origin with less mutation acorss the evolutionary lineage. FTIR, LC-ESI-MS and NMR spectroscopic analysis characterized this purified compound as mycosterol. By comparing with the previous reports, compound was identified as mycosterol^23–25^. The MS/MS spectra and the proposed fragmentation pattern of mycosterol is shown in Fig. 5A. The IC_50_ value of sub fraction of chloroform of *G*. *sylvestre* against α-amylase is 10.47 ± 0.0005 µg/mL and it is better as compared to extract of leaves of *Petalostigma banksii* and *P*. *pubescens* (IC_50_ value of 166.50 ± 5.50 μg/mL and 160.20 ± 27.92 μg/mL, respectively)^26^ and also from methanolic extract of *Phyllanthus virgatus* (IC_50_ value of 33.20 ± 0.556 *μ*g/mL)^27^.

The bioactive compound of *F*. *equiseti* exhibited significant inhibitory activity against α-amylase with IC_50_ value 4.22 ± 0.0005 µg/mL and it is more potent than *Alternaria longipes* strain VITN14G isolated from *Avicennia officinalis* (IC_50_ value of 27.05 µg/mL)^28^. The IC_50_ value of sub fraction of chloroform of *G*. *sylvestre* against α-glucosidase is 85.73 ± 0.001 µg/mL and it is better as compared to extract of leaves of *Petalostigma pubescens* (IC_50_ value of 167.83 ± 23.82 μg/mL)^26^ and ethyl acetate fraction of *Cornus capitata* (IC_50_ value of 50 μg/mL)^29^. The bioactive sub fraction derivative of *F*. *equiseti* exhibited significant inhibitory activity against α-glucosidase with IC_50_ value 69.72 ± 0.001 µg/mL and is better than ethyl acetate fraction of *Phlomis tuberose* (IC_50_ value of 100 μg/mL)^30^. Result of MTT assay also showed that potent compound is non-cytotoxic.

*In-silico* study showed that mycosterol binds to the binding site of α-amylase and α-glucosidase similar to that of acarbose but with high affinity. The prominent feature of α-amylase is the presence of three extremely conserved and catalytic residues such as Asp197, Asp300 and Glu233 in the active site pocket. In previous study, similar types of hydrophobic interactions and hydrogen bonding have been found in crystal structures of α-amylase with different inhibitor molecules. In 2018, Sohretoglu *et al*. suggested that hydroxyl group of inhibitor increases the inhibition activity^31^. In this study, the isolated compound possess a hydroxyl group at the C-3 position of first carbon ring which may increase the activity of α-amylase and α-glucosidase inhibition. Computational docking analysis also revealed that compound is competitive inhibitor for α-amylase. Its binding conformation was similar to that of acarbose. The synthetic steroidal drug tibolone is transformed by fungus *Fusarium lini*, which inhibit α-glucosidase^32^. Similarly this *F*. *equiseti* may also be transformed to synthesize potent antidiabetic bioactive sub fraction derivative on large scale in lesser span of a time. Additionally, recently two glibenclamide-pregnenolone derivatives with hypoglycaemic activity were prepared^33^.

## Conclusion

After isolation of fungal endophytes from *G*. *sylvestre,* the comprehensive screening to find the potential fungal endophytes having antidiabetic activity as α-amylase and α-glucosidase inhibitior was carried out. In this study, we have isolated and identified potent endophytic fungal species *F. equiseti* from the leaves of *G*. *sylvestre*. The bioactive compound was isolated and identified by FTIR, LC-ESI-MS, ^1^H NMR and ^13^C NMR. This mycosterol showed the similar characteristic appeared in phytosterol such as β-sitosterol. The known bioactive compound mycosterol was authenticated by comparing their NMR data with those of reported previously. This isolated mycosterol, exhibited significant α-amylase and α-glucosidase inhibition activity. Kinetics study also confirmed the competitive mode of inhibition to α-amylase and uncompetitive mode of inhibition to α-glucosidase. The biopharmaceutical importance of extract of *F. equiseti* (MF 403109) was further established by the cytotoxic activity against mice fibroblast cell line (L929) ; our MTT results indicated that it is safe even at high doses. *In-silico* studies revealed that mycosterol is a competitive inhibitor for α-amylase and α-glucosidase and binds to the active site similar to that of acarbose but with high affinity due to the presence of hydroxyl group at the C-3 position of the first carbon ring of the compound.

## Methods

### Collection of plant material

The medicinal plant, *G. sylvestre* was harvested from the Barkachha campus of Banaras Hindu University, Mirzapur, U.P., India for study. Healthy tissues (leaves, roots and stem) of four plants (2–3 years old) were collected randomly and were cut into small pieces (1.0 × 1.0 cm), samples were and kept in the sterile polythene bags. All the samples were screened for endophytic fungi within 2 days of collection.

### Isolation and culture of endophytes

All small pieces (1.0 × 1.0 cm) were washed under running tap water and were dried under aseptic conditions. The margin of small pieces of leaves, roots and stems were cut and initially surface treated with 70% ethanol for 1 min to eliminate the epiphytic microorganisms. Thereafter, sterilization of tissue with aqueous sodium hypochlorite (4% available chlorine) for 3 min and then rinsed in 70% ethanol for nearly 30 seconds. before a final triple rinsing in sterilized double distilled water, later the tissue was air dried. The concentration of 100 µg/mL of streptomycin was added in potato dextrose agar (PDA) media to prevent the bacterial contamination. Each culture plate contain four segments of tissue and plates were sealed with parafilm and was incubated in a BOD incubator in 12-hours light and dark cycle at 27 ± 2 °C for 1 week. After 1 week, actively growing hyphal tips of fungi were then sub cultured into new PDA media.

### Extraction and purification of metabolites of endophytic fungi and *G. sylvestre*

The pure strain of isolated fungi was grown in 500 ml erlenmeyer flasks containing 200 ml potato dextrose broth and incubated in BOD cum orbital shaker for 2 weeks at 28 ± 2 °C with periodical shaking at 240 rpm. The mycelium of fungal culture was removed by filtration and broth was then extracted gradually with heptanes, chloroform and ethyl acetate in separating funnel for three times. The organic phase was evaporated to dryness under reduced pressure using a rotary evaporator to constitute the crude broth extract. The crude extract was lypholized in lypholizer and was stored at −20 °C.

The dried leaves of *G*. *sylvestre* were grinded into powder and extracted separately with 500 ml of four different solvents i.e. heptane, chloroform and ethyl acetate in a soxhlet extractor for 6 hours. The organic solution was evaporated to dryness using a rotary evaporator and then crude extracts were dissolved in 90% methanol for screening of α-glucosidase and α-amylase inhibition. The active extract was fractionated by silica gel vacuum liquid chromatography (60–120) using a successive stepwise gradient of chloroform: acetone (10:90, 20:80, 40:60, 70:30) and absolute chloroform. Isolated active fraction (i.e. 40:60) was further sub fractionated by HPTLC with two different mobile phases. Firstly, it was run with mobile phase-I i.e. toluene: acetone: ethyl acetate: 25% of ammonia (10:10:1.5:0.5) and then in mobile phase-II i.e. chloroform: benzene: methanol: formamide (2:20:5:0.250). Similarly, chloroform soluble extract of *F*. *equiseti* was run into two different mobile phases. Firstly in benzene: acetone (4:1) and then in toluene: ethyl acetate: formic acid: methanol (6:6:1.8:0.25).

### Morphological identification of the endophytic fungal Isolates

The endophytic fungi were identified according to their macroscopic characteristics such as colony morphology and spore morphology under Light microscope and also by Scanning Electron Microscopy (SEM). Each identified endophytic fungus was assigned with specific code numbers and maintained in cryo-vials on Potato Dextrose Agar media layered with glycerol (15%,v/v) and also in a lyophilized form. All Fungal strains were stored at −20 °C for future use.

### Isolation of total genomic DNA

The culture plate was washed with 1X PBS, 1 mL of RiboZol per 10 cm^2^ of culture disc area was added further. The cells were lyzed by continuous pipetting and all substance were transferred into a nuclease free tube. The cells were homogenized and were incubated for 5–10 minutes at RT. 200 µL of chloroform per 1 mL RiboZol was added, tube was further shaken vigorously for 15 seconds to mix the sample, later it was incubated for 2–3 minutes at RT. Sample was centrifuged at 12,000 rpm for 15 minute at 4 °C. Three separated phases were observed. Aqueous phase was removed and 0.3 mL ethanol per mL of RiboZol was added, samples were mixed well and were incubated for 3 minutes at 15–30 °C. Mix was centrifuged at 12,000 rpm for 5 minutes at 4 °C, later the supernatantwas discarded. The bottom of the tube contained DNA which was further washed by adding 0.1 M sodium citrate/10% ethanol (1 mL per ml RiboZol) to the pellet. The resulting mix was further incubated for 30 minutes at 15–30 °C; after that it was centrifuged at 12,000 rpm for 5 minutes at 4 °C. DNA washing step was repated, pellet was resuspended in 75% ethanol, incubated for 10–20 minutes at 15–30 °C with intermittent mixing. Mix was further centrifuged at 12,000 rpm for 5 minutes at 4 °C, pellet was air dried for 5–10 minutes. Pellet was further dissolved in 1X TAE buffer solution and the mix was stored for further experiments.

### DNA sequencing of fungal isolate

Polymerase Chain Reaction (PCR) was performed in a T-100 Thermal Cycler, TTC-100 in a total volume of 25 μL for a selected strain. The PCR mixture contained 4 μL 2X Taq buffer, 0.4 mM deoxynucleotide triphosphate (dNTP), 0.8 μM each primer, 0.75 units of Taq DNA polymerase and 6 ng of template DNA. Two primer ITS1 (5′TCCGTAGGTGAACCTGCGG3′) and ITS2 (5′GCTGCGTTCTTCATCGATGC 3′) with 19 and 20 bases respectively, were used. DNA amplification was performed with an initial denaturation of 1 min at 94 °C followed by 30 cycles at 94 °C for 30 sec, at 59 °C for 55 sec, at 72 °C for 90 sec and a final extension of 1 min at 72 °C. The PCR for ITS regions was performed at 95 °C (2 min) for a hot start, followed by 30 cycles of 94 °C (1 min), 56 °C (30 s), 72 °C (2 min), and a final extension of 72 °C (10 min). PCR products were purified using Montage PCR Clean up kit. Sequencing reactions were performed using a ABI PRISM® BigDyeTM Terminator Cycle Sequencing Kits with AmpliTaq® DNA polymerase (FS enzyme) and were sequenced on a ABI 3730 × l DNA analyzer.

### *In-silico* study for sequencing alignment and phylogenetic analysis

The *in-silico* BLAST tool was used to compare the specificity of the selected PCR primers to amplify *Fusarium equiseti* among other species. The ITS sequences of *Fusarium sp*. of 70 strains were downloaded from the NCBI GenBank database and these were used as reference sequences in the phylogenetic analyses. All these sequences were aligned with the program MUSCLE3.7. The resulting aligned sequences were cured using the program G blocks 0.91b. Finally, the program PhyML3.0 aLRT was used for tree building. The program Tree Dyn198.3 was used for tree rendering. The DNA sequences thus obtained were submitted to the ribosomal gene database (https://ncbi.nim.nih.gov) and the sequences were aligned to identify the fungus. This multiple-alignment file was used for phylogenetic analysis which was performed using Mega 6 with Neighbor-Joining method.

### Fourier transform infra red spectroscopy (FTIR)

 Chemical characterization of potent fraction was done by FTIR, NMR and ESI mass. FTIR is a well-known tool for detection of presence of functional groups. The bioactive subfraction (100 mg) was subjected to FTIR analysis (JASCO 1400, JASCO, Tokyo, Japan). In this analysis, sample was prepared in potassium bromide discs and scanned within the range of 500–4000 cm^−1^. The absorbances of molecular vibrations under IR radiation are proportional to the abundance of the functional groups.

### Liquid Chromatography-Electrospray Ionization-Mass Spectrometry (LC-ESI-MS)

Sample was dissolved in 80% methanol and filtered through 0.45 μm PVDF filters. The LC-MS (LC-MS2010EV, Shimadzu, Japan) analysis was carried out under three mobile phases: in mobile phase A, the Waters Cosmosil 5C18-AR-II column (5 μm, 4.6 × 150 mm) was used with 0.25% methanol and mobile phase B contained acetonitrile while mobile phase C had only water. Ratio of three mobile phase (A:B:C) was kept 20:20:60, gradient elution was run with the flow rate of 1.0 mL/min. For the detection of compound, 5 μL of injection volume and 200 nm of wavelength was used. Mass spectrum of the methanolic extract of potent fraction of *F*. *equiseti* was recorded in LC-MS. The potent fraction was analyzed by full scan of ESI using both positive and negative modes of ionization. The identity and molecular mass of the potent fraction was confirmed by comparing their mass/charges ratio with those on the stored library (Metwin version 2.0, MetLife India Insurance Company, Chennai, India).

### Nuclear magnetic resonance (NMR) spectroscopy

NMR spectrum of compound was obtained on a Bruker (500 MHz) NMR (Bruker, Switzerland) at a constant temperature, controlled and adjusted to room temperature. The chemical shifts were shown in δ values (ppm) with tetra-methylsilane as an internal standard. The deuterated chloroform was used as the solvent for recording of ^1^H and ^13^C NMR spectra.

### α-amylase inhibition assay

The activity of α-amylase was carried out using starch as a substrate as described by Khare and Prakash^34^. Reaction mixture (1.0 ml) containing starch was dissolved in sodium phosphate buffer (100 mM, pH 6.9), suitably diluted porcine pancreatic α-amylase was incubated at 27 °C for 3 min. The reaction was terminated by adding 1.0 ml of 3,5-dinitrosalicylic acid solution followed by heating the reaction mixture in a boiling water bath for 5 min and subsequently cooling down to room temperature. Thereafter, 10 ml of double distilled water was added and the amount of reducing sugar (maltose) produced was quantitated using spectrophotometer at 540 nm. For α-amylase inhibition assay, 250 μl of enzyme dissolved in sodium phosphate buffer (100 mM, pH 6.9) was added to 500 μl of test sample containing plant (10.47 µg/ml) or fungal extract (4.22 µg/ml) concentration respectively, followed by incubation at 37 °C for 30 minutes. To this, 250 μl of starch of varying concentration of 0.25–8 mg/mL dissolved in sodium phosphate buffer (100 mM, pH 6.9) was added. Further, reaction mixture (1 mL) was assayed for enzymatic activity as described under standard assay conditions. In case of control, inhibitor was not added to the reaction mixture and the enzyme assay was carried out accordingly. Acarbose (5.75 µg/ml) was used as the reference standard inhibitor.

### α- glucosidase inhibition assay

The α-glucosidase inhibition test was carried out in 96-well microplate using a modified procedure of Bilal *et al*.^35^. The reaction mixture contained 50 μL of sodium phosphate buffer (100 mM, pH 6.8), 10 μL of α-glucosidase and 20 μL of plant (85.73 µg/ml)/fungal extract (69.72 µg/ml) as test sample; samples were thoroughly mixed in a 96-well microplate and were incubated at 37 °C for 30 minutes. However, in case of control, inhibitor was not added. The reaction was initiated by adding 20 μl of pNPG of varying concentration of 0.25–8 mg/mL, prepared in sodium phosphate buffer (100 mM, pH 6.8) as a substrate and incubated for another 15 minutes at 37 °C. The reaction was terminated by adding 50 μl of sodium carbonate. The absorbance was recorded at 405 nm by a 96-well micro plate reader (Bio-Rad, India). Acarbose (55.29 µg/ml) was used as a reference standard inhibitor.

The percentage of inhibition of both enzymes was calculated using the following equation:

$${\rm{Percentage}}\,{\rm{of}}\,{\rm{enzyme}}\,{\rm{Inhibition}}=({{\rm{A}}}_{{\rm{control}}}-{{\rm{A}}}_{{\rm{sample}}}/{{\rm{A}}}_{{\rm{control}}})\times 100$$

where A_control_ is the activity of enzyme without the inhibitor and A_sample_ is the enzyme activity with the test sample solution as inhibitor at different concentrations. The amount of the inhibitor required for inhibiting 50% of the enzyme activity under the standard assay conditions was used to determine IC_50_ value.

### Kinetics of inhibition against α-glucosidase

The mode of inhibition of the bioactive fractions of plant and fungal extracts against α-amylase and α-glucosidase activity were determined using Lineweaver-Burk plot analysis with increasing concentrations of substrate i.e. starch and p-nitrophenyl-α-D-glucopyranoside in the absence (control) and presence of the inhibitor respectively.

### Cytotoxic assay of mycosterol

The L929 cells were cultured in Dulbecco’s modified Eagle’s medium (DMEM) supplemented with 5% fetal bovine serum (FBS) under a humidified atmosphere of 5% CO_2_ and 95% air at 37 °C. The cytotoxicity of the bioactive fractions of plant and fungal extracts was assayed using L929 cells, mouse fibroblast cell line by MTT (3-[4,5-dimethyl thiazol-2-yl]-2,5-diphenyl tetrazolium bromide) method. A fixed volume of 100 μL, with a density of 2 × 10^4^ cells/ml cell suspension was seeded into each well of 96-well microplate plates, after 24 hours the old media with cell debris was replaced with fresh media, extracts and pure fractions were added in different concentrations in triplicate, incubated for 48 hours. The negative control for the experiment was untreated cells, while the cells treated with 10% DMSO were used as a positive control. Further, the culture media was replaced with 50 μL of MTT solution (0.5 mg/mL) in each well and was incubated for 4 hour at 37 °C, followed by removalof supernatant and solubilisation of water insoluble violet formazan crystals in 100 μL of DMSO in each well. The absorbance was measured by microplate reader (Bio-Rad, India) at 570 nm^36,^ results are shown in Fig. 9A,B.

### Statistical analysis

The data represented in the study are the mean values of three replicates (n = 3) and shown as means ± standard deviation (SD). One Way Anova was used for comparison between groups (p < 0.05). The customized module of GraphPad was used for IC_50_ values through nonlinear regression, comprising a dose-response inhibition.

### *In-silico* study of bioactive fraction of fungal extract against α-amylase and α-glucosidase enzymes

In molecular docking analysis, the SDF file of potent fraction was converted into PDB (PDB ID: 1HNY and 5NN3) by using the ChemDraw software. The molecular docking study of compound was done by using PatchDock online server. The results obtained by PatchDock were ranked according to geometric shape, surface patch matching and complementarily score after molecular shape representation. For visualisation and determining the mode of interaction between the receptor and ligands, Discovery Studio 4.0 Client was used^37^.
